# Multimodal prognostic models for bladder urothelial carcinoma: uroplakin III combined with serum and demographic data

**DOI:** 10.3389/fonc.2025.1636358

**Published:** 2025-09-19

**Authors:** Runlin Feng, Jian Hou, Yanping Tao, Yumin Wang, Songzhou Li, Xingyuan Dong, Wenlin Tai

**Affiliations:** ^1^ Department of Pathology, The Second Affiliated Hospital of Kunming Medical University, Kunming, Yunnan, China; ^2^ Department of Urology, The First Affiliated Hospital of Kunming Medical University, Kunming, Yunnan, China; ^3^ Department of Emergency Medicine, Kunming Third People’s Hospital, Kunming, Yunnan, China; ^4^ Department of Laboratory Medicine, The Second Affiliated Hospital of Kunming Medical University, Kunming, Yunnan, China

**Keywords:** BUC, UPK3A protein, prognostic prediction, ML, serum inflammatory markers, risk stratification, shap, multimodal data

## Abstract

**Background:**

Bladder urothelial carcinoma (BUC) remains a highly recurrent and heterogeneous malignancy. Accurate postoperative risk stratification is crucial to guide adjuvant therapy decisions. We hypothesized that integrating Uroplakin III (UPK3A protein)protein expression with systemic inflammation markers and demographic factors could improve prognostic prediction through advanced machine learning(ML) models.

**Methods:**

This retrospective study analyzed 1,032 BUC patients who underwent radical cystectomy. Clinical, pathological, and serological data, including immunohistochemical UPK3A protein expression, were collected. Least Absolute Shrinkage and Selection Operator (LASSO) regression with λ=0.009 (determined via 10-fold cross-validation) was used for feature selection. Nine ML models were trained and validated. Model performance was assessed using Area Under the Receiver Operating Characteristic Curve (AUC-ROC), calibration curves, decision curve analysis (DCA), and clinical impact curves (CIC). Model interpretability was evaluated with SHapley Additive exPlanations (SHAP).

**Results:**

Light Gradient Boosting Machine(LightGBM), Random Forest(RF), and Extreme Gradient Boosting (XGBoost) models demonstrated superior performance (AUCs: 0.894/0.754 for RF in training/test sets). SHAP analysis highlighted vascular invasion, tumor necrosis, and UPK3A protein as key predictors. CIC demonstrated strong clinical utility. Integrating UPK3A protein with inflammatory and demographic variables outperformed traditional models.

**Conclusions:**

The combination of UPK3A protein expression with multimodal features significantly enhances prognostic modeling in BUC. This approach offers a promising clinical decision support tool to stratify risk and guide postoperative management. Future studies should incorporate transcriptomic/proteomic data to further validate these findings.

## 1 Introduction

BUC is one of the most prevalent malignancies in the urinary system, with high recurrence, progression rates, and significant variability in prognosiss (1). While radical cystectomy remains the standard treatment for muscle-invasive bladder cancer (MIBC) and high-risk non-muscle invasive bladder cancer (NMIBC), a substantial proportion of patients experience recurrence or metastasis after surgery, leading to marked differences in outcomes (2). Hence, precise postoperative risk prediction is critical for tailoring patient management and informing adjuvant therapy decisions.

Traditional prognostic assessments primarily rely on tumor staging, pathological grading, vascular invasion, and lymph node metastasis. However, such models often overlook the combined impact of tumor molecular characteristics and host-specific factors (3). Recently, UPK3A, a structural protein specifically expressed on the membrane of urothelial cells, has gained widespread use in bladder cancer diagnostic research. Increasing evidence suggests that UPK3A protein expression not only holds significant diagnostic value but may also be closely associated with the invasiveness, progression, and prognosis of bladder cancer (4, 5). Additionally, serum markers (such as white blood cell count, albumin levels, and urine analysis parameters) reflect the host’s systemic inflammatory state and immune response, which are also recognized to play critical roles in bladder cancer prognosis (6–8). Demographic factors, including age, gender, and smoking history, as basic clinical information, also influence tumor development and patient survival outcomes (9, 10).

In recent years, ML techniques have demonstrated powerful capabilities in constructing medical prediction models by integrating multidimensional, complex data features and capturing underlying patterns that traditional statistical methods may miss (11, 12). Tree-based algorithms, such as XGBoost, random forest (RF), and LightGBM, have shown superior performance in cancer prognosis prediction (13–15).However, there is a lack of comprehensive prognostic models based on the integration of UPK3A protein, serum markers, and demographic features, and systematic studies on their clinical application value and interpretability remain limited.

Therefore, this study was conducted using a large cohort of bladder cancer patients from two affiliated hospitals of Kunming Medical University. We systematically collected data on UPK3A protein expression levels, serum markers, and demographic features, and employed LASSO regression for feature selection. A personalized prognostic prediction model was developed using various ML algorithms, including XGBoost, RF, and LightGBM. Model performance was evaluated through ROC curves, calibration plots, DCA, and CIC, with SHAP analysis enhancing model interpretability. The aim of this study was to explore the potential value of UPK3A protein combined with multiple parameters for predicting prognosis in BUC, facilitating precise postoperative risk assessment and the development of individualized management strategies.

## 2 Materials and methods

The methodology consists of three components: data preprocessing and feature extraction (Section 2.1), model construction and validation (Section 2.2), and reproducibility documentation and implementation environment (Section 2.3).

### 2.1 Data collection and processing

We collected inpatient data from 1,764 patients diagnosed with bladder cancer and undergoing radical cystectomy at the First Affiliated Hospital and the Second Affiliated Hospital of Kunming Medical University between 2014 and 2024. The dataset included demographic characteristics (gender, age, ethnicity, weight, smoking, alcohol consumption, etc.), medical history (hypertension, diabetes, hematuria, frequency, urgency, dysuria, difficulty in urination, and previous surgeries), tumor morphological features (tumor shape, diameter, location, number, presence of a base, boundary clarity, color, texture, presence of necrosis, bleeding, and cystic lesions), as well as pathological features assessed by immunohistochemistry, including UPK3A, GATA Binding Protein 3 (GATA3), Cytokeratin 20 (CK20), Cytokeratin 7 (CK7), Cytokeratin 5/6 (CK5/6), Tumor Protein 63 (P63), Tumor Protein 53 (P53), Androgen Receptor (AR), Programmed Death-Ligand 1 (PD-L1), microsatellite stability, Human Epidermal Growth Factor Receptor 2 (HER2), nerve invasion, vascular invasion, pathological staging, grading, positive surgical margins, and histological types such as squamous, glandular, neuroendocrine, and sarcomatoid.

Inclusion criteria for participants were as follows: (1) patients aged over 18 years; (2) diagnosis of bladder cancer according to the WHO Classification of Tumors of the Urinary and Male Genital Systems (4th Edition), and receipt of radical cystectomy; (3) complete clinical data, including blood count, biochemical tests, pathological parameters, and immunohistochemistry; (4) detailed treatment history with complete follow-up data and results; (5) no prior radiotherapy, chemotherapy, or immunotherapy.

Patients who met any of the following criteria were excluded from the study: (1) patients who underwent partial bladder tumor resection; (2) post-operative pathology confirmed non-urothelial carcinoma; (3) incomplete clinical data or lost to follow-up with no available prognostic data; (4) preoperative radiotherapy, chemotherapy, or immunotherapy; (5) other malignancies metastasized to the bladder; (6) patients under 18 years of age; (7) patients with survival time less than 1 month. The study was approved by the Ethics Committees of the First Affiliated Hospital and the Second Affiliated Hospital of Kunming Medical University, with informed consent obtained from all patients.

To minimize the impact of missing data on model construction, we used the K-Nearest Neighbors (KNN) Imputer method to impute missing data (less than 20% missing), while data with more than 20% missing were excluded. The primary endpoint was the response to postoperative adjuvant therapy, as recorded in the patients’ follow-up treatment records. Missing values were imputed using the `KNNImputer` algorithm (version 0.24.2, scikit-learn), with the number of neighbors set to 5. Variables with more than 20% missing data were excluded from model construction to reduce bias. Continuous variables were standardized using z-score normalization, and categorical variables were encoded using one-hot encoding prior to model input.

### 2.2 Statistical analysis and model construction and validation

Categorical variables are presented as percentages (%) and compared between groups using Pearson’s chi-square test. Due to the imbalance in the dependent variable categories, an undersampling method was applied to resample the data and balance the distribution. A five-fold cross-validation was used to split the dataset into training and internal validation sets. In the case of high-dimensional features, LASSO regression was employed for feature selection. This method applies L1 regularization to shrink regression coefficients, reducing dimensionality, selecting the most informative variables, and eliminating redundant features.

Nine ML algorithms were used for predictive modeling, including XGBoost, support vector machine (SVM), multilayer perceptron (MLP), KNN, logistic regression, LASSO, decision tree (DT), gradient boosting machine (GBM), and RF. All models incorporated the features selected by LASSO. A single cross-validation was performed to ensure model stability. Grid search optimization was applied to fine-tune hyperparameters, and the model with the highest area under the AUC-ROC curve was selected as the optimal model. The final model was constructed on the training set and validated on both internal and external validation sets. Model performance was evaluated using AUC-ROC, sensitivity, specificity, recall, F1 score, and accuracy. All machine learning models were implemented using Python 3.8 with `scikit-learn` (v0.24.2), `xgboost` (v1.5.0), `lightgbm` (v3.3.1), and `shap` (v0.41.0). LASSO regression for feature selection was performed using `LassoCV` from `scikit-learn`, with 10-fold cross-validation to determine the optimal lambda value (λ = 0.009), minimizing binomial deviance. Model hyperparameters (e.g., learning_rate, n_estimators, max_depth) were tuned using `GridSearchCV` with five-fold cross-validation. The hyperparameter configurations for each model are provided in **Supplementary Table S1**.

**Figure 1:**
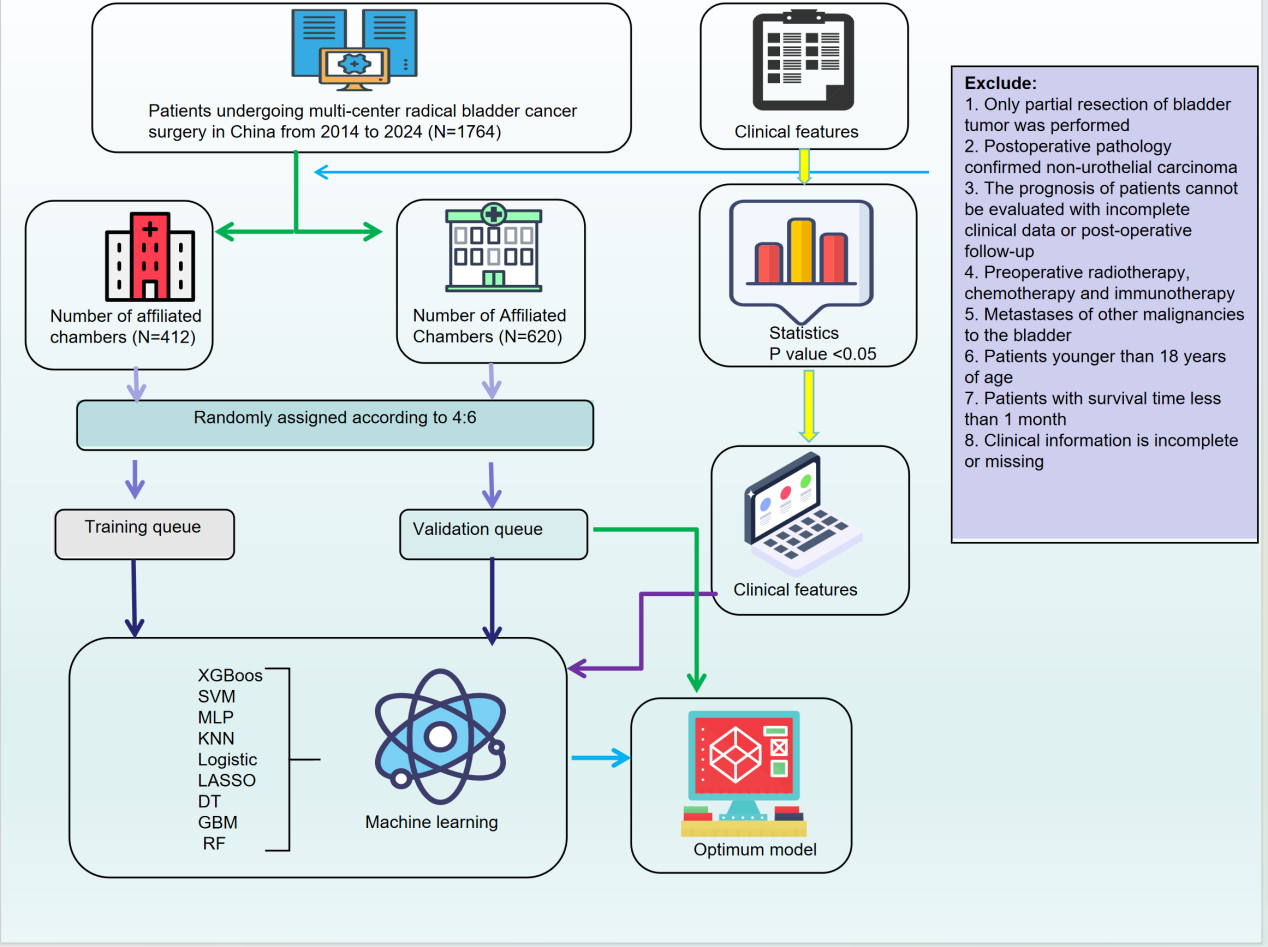
Workflow of model development and validation.

Additionally, to assess the real clinical utility of the model, DCA and calibration curves were plotted. To identify the optimal clinical decision threshold, a clinical impact curve (CIC) was constructed to visually assess the most effective decision threshold. The threshold was derived using the “surv_cutpoint” function in the survminer R package to maximize survival difference. To analyze the impact of the selected features on the model predictions, SHAP analysis was used. SHAP summary plots were generated to show the contribution of each feature to the prediction results, and specific cases were evaluated using SHAP to illustrate the degree of impact of selected features on the predictions. All statistical analyses were conducted in Python, with two-sided p-values < 0.05 considered statistically significant.

(The flowchart of this research is shown in **Figure 1**)

### 2.3 Model reproducibility and technical implementation

All computational procedures were implemented using Python 3.8. Machine learning models, including LightGBM, XGBoost, Random Forest, SVM, and others, were built using the scikit-learn (v0.24.2), xgboost (v1.5.0), and lightgbm (v3.3.1) packages. Data imputation was performed using KNNImputer with default parameters (n_neighbors=5), and variables with more than 20% missing data were excluded. Continuous variables were standardized using z-score normalization, and categorical variables were transformed by one-hot encoding.

LASSO regression for feature selection was conducted using LassoCV from scikit-learn with 10-fold cross-validation to determine the optimal penalty parameter (λ = 0.009), based on minimum binomial deviance. Model hyperparameters were optimized via GridSearchCV with five-fold internal cross-validation. The detailed hyperparameter settings for each model are provided in **Supplementary Table S1**.

Model performance was evaluated using metrics such as accuracy, sensitivity, specificity, precision, negative predictive value (NPV), F1-score, Youden index, and AUC-ROC. All metrics were computed using functions from scikit-learn.metrics. To interpret feature contributions, SHAP (SHapley Additive exPlanations) values were calculated using the TreeExplainer module from the shap Python package (v0.41.0). Both global summary plots and individual force plots were generated to visualize model decision logic and feature importance.

All analytical pipelines, including data preprocessing, model training, evaluation, and SHAP analysis, were version-controlled and archived. The complete source code and training-validation splits are available from the corresponding author upon reasonable request, ensuring full reproducibility.

## 3 Result

### 3.1 Lasso regression for key variable selection and optimization of BUC prognostic prediction model

The complete analytical process of this study is illustrated in **Figure 1**. In this study, based on data from 1,674 bladder cancer patients at two affiliated hospitals of Kunming Medical University, a final cohort of 1,032 eligible cases was included. These cases were randomly divided into a training cohort (N=412) and a validation cohort (N=620) in a 4:6 ratio. Univariate analysis identified clinical features associated with patient outcomes, including age, urinary urgency, dysuria, tumor necrosis, perineural invasion, vascular invasion, tumor diameter, tumor location, tissue texture, as well as several blood and urine markers (such as creatinine, neutrophil count, and leukocyte esterase), which showed significant differences between outcome groups (P<0.05) (**Table 1**).

**Table 1:** Baseline Uroplakin III, serum and demographic date in bladder.

Characteristic	0 N = 410^1^	1 N = 624^1^	p-value^2^
Gender			0.7
1	347 (85%)	534 (86%)	
2	63 (15%)	90 (14%)	
Age	230 (56%)	310 (50%)	0.043
Ethnicity	63 (15%)	77 (12%)	0.2
Smoking	215 (52%)	292 (47%)	0.076
History of Prior Surgery	88 (21%)	106 (17%)	0.071
Alcohol Consumption	128 (31%)	187 (30%)	0.7
Diabetes	44 (11%)	69 (11%)	0.9
Frequent Urination	146 (36%)	196 (31%)	0.2
Urinary Urgency	115 (28%)	171 (27%)	0.8
Dysuria	76 (19%)	86 (14%)	0.040
Pain	91 (22%)	109 (17%)	0.060
Urinary Hesitancy	65 (16%)	83 (13%)	0.3
Red Blood Cells in Urine	393 (96%)	592 (95%)	0.5
White Blood Cells in Urine	383 (93%)	580 (93%)	0.8
Epithelial Cells in Urine	143 (35%)	232 (37%)	0.5
Crystals in Urine	15 (3.7%)	28 (4.5%)	0.5
Urinary Cytology	46 (11%)	49 (7.9%)	0.067
Bacteria in Urine	24 (5.9%)	87 (14%)	<0.001
Perineural Invasion	187 (46%)	108 (17%)	<0.001
Vascular Invasion	251 (61%)	163 (26%)	<0.001
M	62 (15%)	12 (1.9%)	<0.001
CIS	44 (11%)	46 (7.4%)	0.061
Surgical Margins	51 (12%)	43 (6.9%)	0.002
Urop0III	270 (66%)	505 (81%)	<0.001
Number of Tumors	144 (35%)	284 (46%)	<0.001
Well-defined Borders	143 (35%)	304 (49%)	<0.001
Necrosis	172 (42%)	143 (23%)	<0.001
Hemorrhage	106 (26%)	141 (23%)	0.2
Cystic Degeneration	12 (2.9%)	14 (2.2%)	0.5
Duration of dringking			0.8
0	282 (69%)	437 (70%)	
1	33 (8.0%)	56 (9.0%)	
2	25 (6.1%)	35 (5.6%)	
3	70 (17%)	96 (15%)	
Blood Pressure			0.7
0	309 (75%)	474 (76%)	
1	84 (20%)	117 (19%)	
2	12 (2.9%)	20 (3.2%)	
3	5 (1.2%)	13 (2.1%)	
Hematuria			0.047
0	77 (19%)	89 (14%)	
1	12 (2.9%)	32 (5.1%)	
2	321 (78%)	503 (81%)	
Duration of Smoking			0.2
0	195 (48%)	332 (53%)	
1	51 (12%)	80 (13%)	
2	63 (15%)	78 (13%)	
3	101 (25%)	134 (21%)	
Total Protein			0.6
0	163 (40%)	261 (42%)	
1	241 (59%)	357 (57%)	
2	6 (1.5%)	6 (1.0%)	
Albumin			>0.9
0	144 (35%)	214 (34%)	
1	266 (65%)	409 (66%)	
2	0 (0%)	1 (0.2%)	
Globulin			0.11
0	227 (55%)	360 (58%)	
1	108 (26%)	131 (21%)	
2	75 (18%)	133 (21%)	
Albumin/Globulin Ratio			0.2
0	147 (36%)	194 (31%)	
1	258 (63%)	418 (67%)	
2	5 (1.2%)	12 (1.9%)	
Total Bilirubin			0.12
0	299 (73%)	417 (67%)	
1	4 (1.0%)	7 (1.1%)	
2	107 (26%)	200 (32%)	
Urea			0.013
0	264 (64%)	432 (69%)	
1	24 (5.9%)	53 (8.5%)	
2	122 (30%)	139 (22%)	
Creatinine			<0.001
0	280 (68%)	470 (75%)	
1	8 (2.0%)	26 (4.2%)	
2	122 (30%)	128 (21%)	
Uric Acid			0.12
0	321 (78%)	464 (74%)	
1	25 (6.1%)	60 (9.6%)	
2	64 (16%)	100 (16%)	
White Blood Cells			0.004
0	282 (69%)	485 (78%)	
1	17 (4.1%)	24 (3.8%)	
2	111 (27%)	115 (18%)	
Neutrophils			0.010
0	248 (60%)	432 (69%)	
1	8 (2.0%)	14 (2.2%)	
2	154 (38%)	178 (29%)	
Lymphocytes			0.5
0	292 (71%)	459 (74%)	
1	112 (27%)	160 (26%)	
2	6 (1.5%)	5 (0.8%)	
Monocytes			>0.9
0	357 (87%)	545 (87%)	
1	16 (3.9%)	24 (3.8%)	
2	37 (9.0%)	55 (8.8%)	
Hemoglobin			0.045
0	130 (32%)	245 (39%)	
1	271 (66%)	365 (58%)	
2	9 (2.2%)	14 (2.2%)	
Platelets			0.5
0	285 (70%)	454 (73%)	
1	26 (6.3%)	33 (5.3%)	
2	99 (24%)	137 (22%)	
Occult Blood in Urine			0.061
0	34 (8.3%)	37 (5.9%)	
1	36 (8.8%)	52 (8.3%)	
2	86 (21%)	101 (16%)	
3	254 (62%)	434 (70%)	
Ketones in Urine			0.2
0	398 (97%)	590 (95%)	
1	9 (2.2%)	26 (4.2%)	
2	3 (0.7%)	8 (1.3%)	
Nitrites in Urine			0.6
0	391 (95%)	588 (94%)	
1	10 (2.4%)	16 (2.6%)	
2	9 (2.2%)	20 (3.2%)	
Urine Protein			0.7
0	101 (25%)	153 (25%)	
1	171 (42%)	274 (44%)	
2	138 (34%)	197 (32%)	
Leukocyte Esterase in Urine			<0.001
0	350 (85%)	452 (72%)	
1	28 (6.8%)	78 (13%)	
2	22 (5.4%)	61 (9.8%)	
3	9 (2.2%)	30 (4.8%)	
4	1 (0.2%)	3 (0.5%)	
Glucose in Urine			0.9
0	391 (95%)	595 (95%)	
1	8 (2.0%)	15 (2.4%)	
2	4 (1.0%)	6 (1.0%)	
3	2 (0.5%)	4 (0.6%)	
4	5 (1.2%)	4 (0.6%)	
Urobilinogen in Urine			0.2
0	396 (97%)	607 (97%)	
1	12 (2.9%)	10 (1.6%)	
2	2 (0.5%)	7 (1.1%)	
N			<0.001
0	253 (62%)	546 (88%)	
1	75 (18%)	49 (7.9%)	
2	45 (11%)	18 (2.9%)	
3	37 (9.0%)	11 (1.8%)	
Tumor Diameter			0.005
1	20 (4.9%)	43 (6.9%)	
2	151 (37%)	285 (46%)	
3	145 (35%)	191 (31%)	
4	94 (23%)	105 (17%)	
Location			0.005
0	84 (20%)	119 (19%)	
1	25 (6.1%)	20 (3.2%)	
2	16 (3.9%)	26 (4.2%)	
3	141 (34%)	175 (28%)	
4	144 (35%)	284 (46%)	
Tumor Shape			0.091
0	278 (68%)	468 (75%)	
1	33 (8.0%)	37 (5.9%)	
2	35 (8.5%)	41 (6.6%)	
3	64 (16%)	78 (13%)	
Color			0.6
0	211 (51%)	303 (49%)	
1	97 (24%)	159 (25%)	
2	102 (25%)	162 (26%)	
Texture/Consistency			<0.001
0	69 (17%)	158 (25%)	
1	252 (61%)	395 (63%)	
2	89 (22%)	71 (11%)	

^1^n (%).

^2^Pearson's Chi-squared test; Fisher's exact test.

**Table 1**: Baseline UPK3A, Serum and Demographic Date in Bladder cancer (Please refer to the attached file (**Table 1**. DOCX) for details).

**Figure 2:**
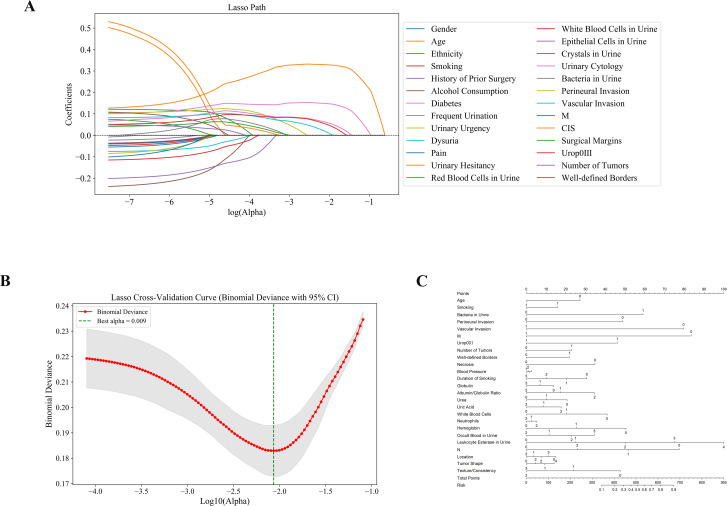
Identification of predictive features using LASSO regression and construction of the nomogram model. **(A)** LASSO coefficient profiles: Displays how the coefficients of 26 features shrink with increasing penalty, identifying key predictors associated with treatment response. **(B)** Cross-validation plot: The optimal lambda (λ = 0.009) was selected using 10-fold cross-validation to minimize binomial deviance. **(C)** Nomogram model: A predictive nomogram was developed based on selected clinical, pathological, and molecular features to estimate individual response probabilities.

Subsequently, clinical variables with statistical significance were selected through univariate analysis, and LASSO regression was applied for further feature selection. The LASSO path plot (**Figure 2A**) shows that as the regularization parameter λ increases, the regression coefficients of some features gradually converge to zero. The cross-validation curve (**Figure 2B**) determined that the optimal λ value was 0.009, which minimized the binomial deviance of the model. The final selected features included age, smoking history, positive urine bacterial culture, perineural invasion, vascular invasion, muscle layer invasion (M stage), UPK3A expression, tumor number, tumor boundary characteristics, and necrosis (**Figure 2C**). These features provided an essential foundation for subsequent model construction.

Among these factors, smoking history, urine leukocytes, and vascular invasion were considered key prognostic factors for BUC, as these features are likely closely related to the mechanisms of cancer development and progression. For example, smoking, a known risk factor for bladder cancer, may contribute to the malignant transformation of urothelial cells through the accumulation of carcinogens, while an increase in urine leukocytes may suggest the role of inflammatory responses in tumor progression.

### 3.2 Construction and performance evaluation of a machine learning-based prognostic prediction model for BUC

This study constructed multiple ML models (KNN, RF, XGBoost (XGB),SVM, Logistic Regression (LR), MLP, LightGBM, LASSO, and DT to predict the prognosis of BUC. The models’ performance was systematically evaluated using ROC curves, Calibration Curves, and DCA to identify the optimal predictive model.

**Figure 3:**
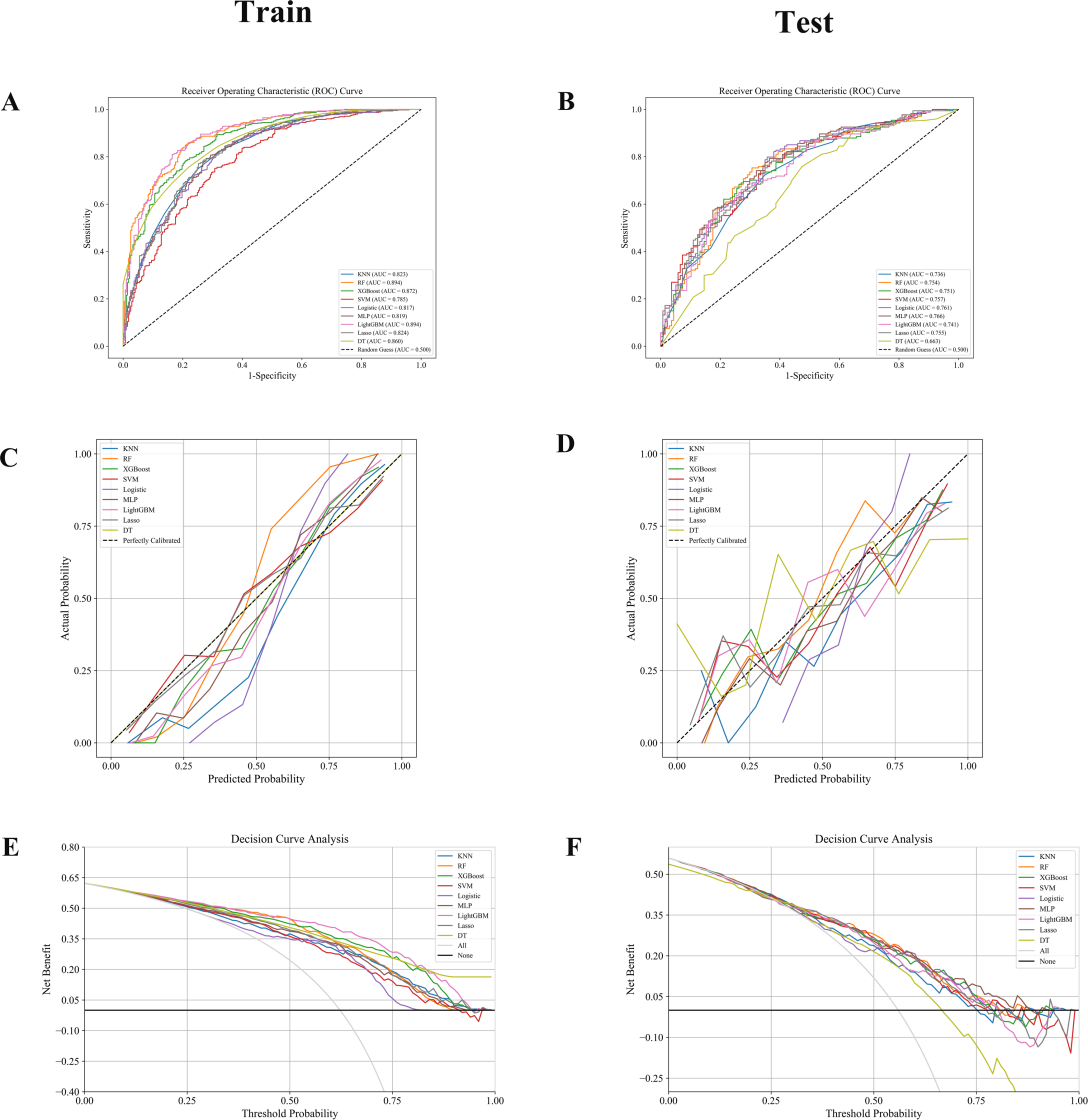
**(A, B)** ROC curves: RF, LightGBM, and XGBoost models achieved superior AUCs, indicating excellent classification performance. **(C, D)** Calibration curves: Good agreement was observed in the training set, while the test set showed greater variability. **(E, F)** DCA: RF and LightGBM consistently provided the highest net benefit across decision thresholds.

Our results indicate that in both the training and validation sets, LightGBM, RF, and XGB models demonstrated excellent predictive performance. The training set AUCs were 0.894, 0.894, and 0.872, respectively (**Figure 3A**, **Table 2**), and the validation set AUCs were 0.741, 0.754, and 0.751, respectively (**Figure 3B**, **Table 2**). LightGBM and RF also outperformed other models in terms of Accuracy, Recall, and F1-Score. The confusion matrix (**Table 3**) further validated the stability of the models in classifying true positives (TP) and true negatives (TN).

**Table 2:** Comparison of prediction performance of nine ML models.

Comparison of results across models										
Train		KNN	RF	XGB	SVM	LR	MLP	LightGBM	Lasso	DT
	Accuracy	0.752	0.824	0.801	0.739	0.726	0.765	0.826	0.77	0.783
	PPV	0.776	0.827	0.802	0.735	0.771	0.766	0.827	0.767	0.781
	Recall	0.752	0.824	0.801	0.739	0.726	0.765	0.826	0.77	0.783
	F1_score	0.727	0.825	0.794	0.727	0.683	0.754	0.821	0.765	0.781
	AUC	0.823	0.894	0.872	0.785	0.817	0.819	0.894	0.824	0.86
	Specificity	0.418	0.799	0.615	0.513	0.315	0.535	0.67	0.604	0.678
	NPV	0.851	0.752	0.812	0.714	0.887	0.772	0.836	0.74	0.728
	Youden Index	0.17	0.623	0.416	0.251	0.041	0.3	0.496	0.375	0.461
	Kappa	0.415	0.631	0.555	0.411	0.333	0.468	0.614	0.493	0.532
Test		KNN	RF	XGB	SVM	LR	MLP	LightGBM	Lasso	DT
	Accuracy	0.678	0.72	0.701	0.714	0.659	0.695	0.688	0.707	0.656
	PPV	0.699	0.722	0.703	0.72	0.691	0.701	0.689	0.709	0.653
	Recall	0.678	0.72	0.701	0.714	0.659	0.695	0.688	0.707	0.656
	F1_score	0.654	0.721	0.693	0.704	0.623	0.682	0.68	0.701	0.65
	AUC	0.736	0.754	0.751	0.757	0.761	0.766	0.741	0.755	0.663
	Specificity	0.401	0.701	0.54	0.533	0.328	0.489	0.526	0.555	0.518
	NPV	0.753	0.676	0.712	0.745	0.763	0.728	0.692	0.717	0.634
	Youden Index	0.08	0.421	0.241	0.247	-0.012	0.184	0.214	0.262	0.174
	Kappa	0.314	0.435	0.377	0.401	0.264	0.358	0.351	0.392	0.288

**Table 3:** Comparison of confusion matrix outputs for nine ML models in the training and testing sets.

Comparison of confusion matrix results across models									
Train					Test				
	TN	FP	FN	TP		TN	FP	FN	TP
KNN	114	159	20	430	KNN	55	82	18	156
RF	218	55	72	378	RF	96	41	46	128
XGB	168	105	39	411	XGB	74	63	30	144
SVM	140	133	56	394	SVM	73	64	25	149
LR	86	187	11	439	LR	45	92	14	160
MLP	146	127	43	407	MLP	67	70	25	149
LightGBM	183	90	36	414	LightGBM	72	65	32	142
Lasso	165	108	58	392	Lasso	76	61	30	144
DT	185	88	69	381	DT	71	66	41	133

The calibration performance of the models was assessed through Calibration Curves, and most models, particularly LightGBM and RF, showed good agreement between predicted probabilities and actual observations in both the training and validation sets (**Figures 3C, D**). DCA (**Figures 3E, F**) further demonstrated that LightGBM, RF, and XGB models provided higher net clinical benefits at various probability thresholds, suggesting that these models have substantial potential for practical clinical application.

In summary, this study constructed and validated a series of machine learning-based prognostic prediction models for BUC. XGBoost and LightGBM exhibited superior performance in classification (AUC), calibration (accuracy of predicted probabilities), and clinical DCA, making them suitable for prognostic prediction and personalized risk assessment in BUC patients. These results provide clinicians with effective risk stratification tools, helping to more accurately identify high-risk patients and formulate individualized treatment strategies.

### 3.3 Clinical application evaluation of the prognostic model for BUC based on CIC

The clinical application value of different ML models in predicting the prognosis of BUC was further evaluated using CIC. CIC primarily illustrates the number of patients predicted as high-risk at various risk thresholds and the number of those who actually experience the target event (e.g., disease recurrence or progression).

The analysis in this study shows that the LightGBM,RF, and XGBoost models were able to accurately identify a higher number of high-risk patients across various risk thresholds. Furthermore, the proportion of actual events (e.g., disease recurrence or progression) occurring among those predicted as high-risk was higher, with the curve trends closely mirroring the actual event occurrence curve, indicating their higher clinical application value. In contrast, the KNN and DT models showed considerable deviation from the actual results at medium and low-risk thresholds, with lower accuracy.

**Figure 4:**
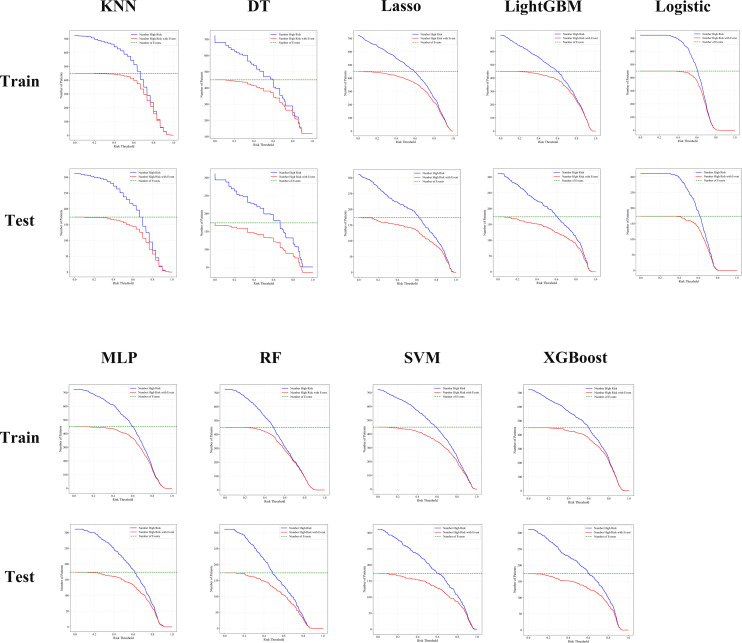
CIC analysis showed that RF, LightGBM, and XGBoost models consistently identified high-risk patients with better accuracy and clinical utility across both training and testing cohorts.

Overall, both RF and LightGBM models maintained a good balance between sensitivity and specificity across all risk thresholds, demonstrating the optimal clinical net benefit. These findings support the use of RF and LightGBM as the preferred models for prognostic risk stratification in BUC patients (**Figure 4**).

### 3.4 Feature contribution assessment of the prognostic model for BUC based on SHAP values

To enhance the interpretability of the model, SHAP analysis was applied in this study. This method quantifies the contribution of each feature to the model’s decision-making process, further exploring its clinical significance. SHAP values reflect the direction and magnitude of each variable’s impact on the model’s output, where positive SHAP values indicate that the variable increases the probability of predicting a high-risk outcome, and negative SHAP values reduce this probability.

**Figure 5:**
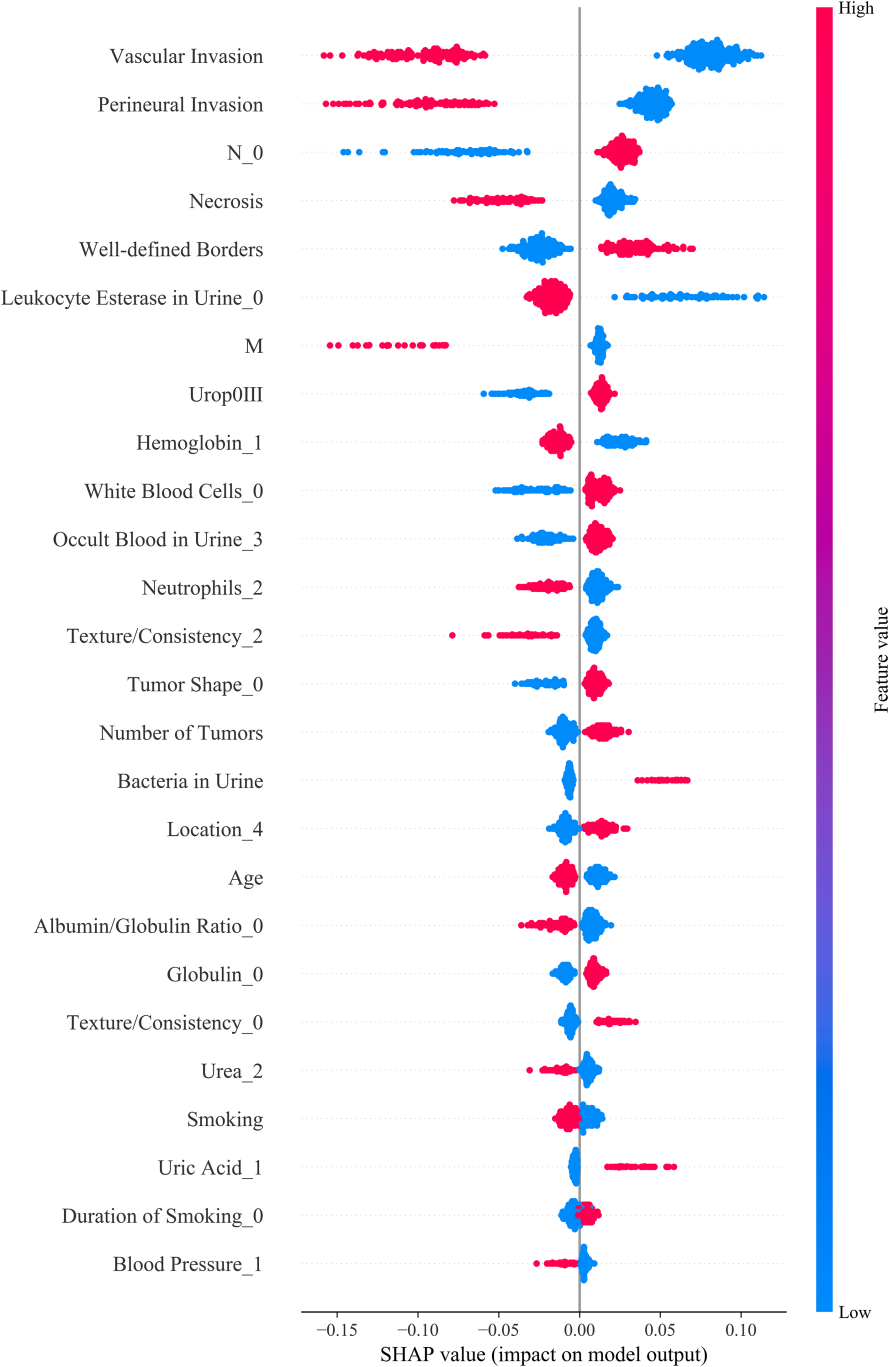
SHAP summary plot illustrating the contribution of each feature to model output. Each dot represents a SHAP value for an individual patient, with color indicating the original feature value (red: high, blue: low). Features are ranked by their mean absolute SHAP values, reflecting their relative importance in predicting prognosis. UPK3A expression, key serum biomarkers (e.g., White Blood Cell), and demographic variables (e.g., Age, Smoking) were among the top contributors. The direction and magnitude of each feature’s impact are visualized, providing insight into how individual predictors influence model decisions.

The analysis revealed that, in the RF model, features such as vascular invasion, perineural invasion, muscle layer infiltration (M stage), tumor necrosis, tumor boundary clarity, urine leukocyte esterase positivity, and white blood cell count had the greatest impact on the model’s predictions. The direction of change in feature values was strongly correlated with the predicted risk levels (**Figure 5**). These findings not only enhance the biological interpretability of the model but also provide a theoretical basis for the postoperative management of bladder cancer patients in the future.

## 4 Discussion

In this study, we developed and validated an interpretable ML–based prognostic model for BUC, integrating UPK3A protein expression with systemic inflammatory markers and demographic data. Our findings support the utility of a multimodal approach to enhance the predictive power of postoperative survival and guide personalized treatment decisions.

In this study, UPK3A protein expression was evaluated at the protein level via immunohistochemical analysis, reflecting its established application in pathological diagnosis rather than gene expression profiling.UPK3A is a transmembrane glycoprotein that plays a pivotal role in maintaining urothelial barrier integrity. Traditionally recognized as a diagnostic marker of urothelial differentiation, recent evidence increasingly supports its role in cancer progression. Several studies have demonstrated that elevated expression of UPK3A protein is associated with advanced tumor stages, aggressive phenotypes, and shorter survival in BUC patients (16–18). In our study, UPK3A protein overexpression, assessed via immunohistochemistry, was independently associated with poor overall survival. These results suggest a potential oncogenic role of UPK3A, possibly via dysregulation of p53 signaling, enhanced proliferation, or immune escape mechanisms.

The prognostic implications of UPK3A may be subtype-specific. In luminal bladder cancer subtypes, UPK3A protein overexpression has been correlated with distinct transcriptional programs and increased resistance to chemotherapy or immunotherapy (19). While UPK3A protein is not yet an established therapeutic target, its cell surface localization renders it an attractive candidate for antibody–drug conjugates (ADCs). Although the current ADC landscape in BUC predominantly focuses on HER2, Nectin-4, and Trop-2, the concept is extensible to other surface proteins such as UPK3A. Recent multicenter real-world studies, including those by Zeng et al. and Zhao et al., have demonstrated promising outcomes using neoadjuvant ADCs (e.g., RC48) in combination with immunotherapy for MIBC (20, 21). These findings highlight the translational potential of surface glycoproteins in guiding targeted therapies.

Beyond molecular markers, we incorporated systemic inflammatory indices such as neutrophil count and leukocyturia. These parameters reflect the host’s systemic immune state and are often indicative of a protumor inflammatory microenvironment. Elevated neutrophil-to-lymphocyte ratio and leukocyturia have been linked to poor prognosis in BUC and may represent surrogates for tumor-promoting inflammation or suppressed antitumor immunity (22–24). Our inclusion of these routinely available laboratory indices adds pragmatic value to the model and facilitates integration into real-world clinical settings.

Model development followed a rigorous pipeline. LASSO regression was used for feature selection with λ = 0.009, followed by ensemble learning using LightGBM, XGBoost, and RF classifiers. All models achieved robust performance with AUCs above 0.74 in validation cohorts. DCA and CIC confirmed clinical utility, and SHAP values revealed that UPK3A protein expression, vascular invasion, and perineural infiltration contributed most to outcome prediction. This interpretability enhances clinical acceptability and transparency, aligning with the growing emphasis on explainable AI in medicine (25, 26).

Importantly, our model adheres to current ESMO recommendations advocating for multi-dimensional risk assessment in urothelial carcinoma (27–29). By integrating tumor biology, host immunity, and clinicopathological variables, our approach surpasses conventional staging systems in granularity and predictive accuracy. Moreover, it supports the vision of precision medicine and data-driven oncology.

Nonetheless, certain limitations must be acknowledged. First, the retrospective design and single-region cohort may limit generalizability. Second, immunohistochemistry does not capture post-translational modifications or alternative splicing variants of UPK3A, which may influence functional outcomes. Third, although UPK3A expression was assessed via immunohistochemistry, the manuscript did not explicitly define the scoring criteria used. In our study, UPK3A staining was semi-quantitatively evaluated using the H-score system, which considers both staining intensity and the percentage of positive cells. However, inter-observer variability remains an inherent limitation of immunohistochemistry-based assessment. Although all immunohistochemistry slides were reviewed independently by two experienced pathologists, no inter-rater concordance coefficient (e.g., kappa statistic) was calculated. Future studies should standardize UPK3A scoring protocols and incorporate digital image analysis or automated quantification to reduce observer-related measurement bias. Fourth, while the model is robust, external multicenter validation in larger cohorts is warranted to confirm reproducibility. Future work should incorporate spatial transcriptomics, single-cell RNA sequencing, and proteogenomic profiling to unravel UPK3A-driven oncogenic networks and treatment resistance (30–32). Additionally, functional studies investigating UPK3A silencing or antibody-mediated blockade may provide critical insights into its therapeutic potential. Finally, our study complements and extends prior real-world research on neoadjuvant therapies in BUC. Zeng et al. and Zhao et al. provided compelling clinical evidence supporting the integration of ADCs and immune checkpoint inhibitors in MIBC (20, 21). Our findings suggest that UPK3A, a luminal lineage marker, may serve as a future therapeutic candidate, particularly in cases unresponsive to conventional therapies. Our comprehensive analysis highlights the multifaceted role of UPK3A in bladder cancer pathogenesis and underscores the importance of incorporating molecular, inflammatory, and clinical data to refine prognostic modeling.

In summary, this study proposes a clinically interpretable and biologically informed prognostic model that underscores the prognostic significance of UPK3A overexpression in BUC. By integrating systemic inflammation, pathological features, and molecular markers, our findings extend previous real-world evidence and offer a foundation for stratified patient management and therapeutic innovation. These results support the future incorporation of UPK3A-guided algorithms into routine prognostic assessment, pending further prospective validation.

## 5 Conclusion

This study presents an interpretable, multimodal prognostic model for postoperative BUC by integrating UPK3A protein expression, systemic inflammatory markers, and clinicopathological features. The model demonstrated favorable predictive accuracy and clinical utility across both internal training and validation cohorts, with LightGBM, Random Forest, and XGBoost achieving optimal performance. Evaluation via AUC-ROC, calibration, DCA, and CIC confirmed its robustness and applicability in clinical decision-making. Notably, the independent prognostic relevance of UPK3A overexpression highlights its potential role as both a biomarker and therapeutic target. While derived from a single-center retrospective cohort, the model offers a pragmatic framework for individualized risk stratification in BUC. Future validation in multi-center, prospective cohorts and incorporation of dynamic and molecular data streams will be essential to further refine and clinically implement this approach.

## Data Availability

The raw data supporting the conclusions of this article will be made available by the authors, without undue reservation.
