# Supplementary material for: Multimodal prognostic models for bladder urothelial carcinoma: uroplakin III combined with serum and demographic data
Source: Front Oncol. 2025 Sep 19;15:1636358. doi: 10.3389/fonc.2025.1636358 (PMC12490981; doi:10.3389/fonc.2025.1636358)
Supplement: Supplementary file 1 [file Table1.docx]

**Supplementary Table S1. Hyperparameter Settings for Machine Learning Models Used in This Study**

| **Model** | **Library/Framework Used** | **Key Hyperparameters** |
| --- | --- | --- |
| LightGBM | lightgbm v3.3.1 | n_estimators=100,learning_rate=0.05, max_depth=6,num_leaves=31, min_child_samples=20,boosting_type='gbdt |
| XGBoost | xgboost v1.5.0 | n_estimators=100, learning_rate=0.05, max_depth=5,subsample=0.8, colsample_bytree=0.8 |
| Random Forest (RF) | scikit-learn v0.24.2 | n_estimators=200, max_depth=8, min_samples_split=2, min_samples_leaf=1, bootstrap=True |
| Support Vector Machine (SVM) | scikit-learn v0.24.2 | kernel='rbf', C=1, gamma='scale', probability=True |
| Multilayer Perceptron (MLP) | scikit-learn v0.24.2 | hidden_layer_sizes=(100,), activation='relu', solver='adam', alpha=0.0001, max_iter=300 |
| k-Nearest Neighbors (KNN) | scikit-learn v0.24.2 | n_neighbors=5, weights='uniform', metric='minkowski', p=2 |
| Logistic Regression (LR) | scikit-learn v0.24.2 | penalty='l2', solver='liblinear', C=1.0, max_iter=100 |
| LASSO | scikit-learn v0.24.2 | alpha=0.01, max_iter=1000, selection='cyclic' |
| Decision Tree (DT) | scikit-learn v0.24.2 | criterion='gini', max_depth=6, min_samples_split=2, min_samples_leaf=1 |
